# Reply to: “Hyperoxemia in postsurgical sepsis/septic shock patients is associated with reduced mortality”

**DOI:** 10.1186/s13054-022-03989-z

**Published:** 2022-06-09

**Authors:** Alessandro N. Franciosi, Cormac McCarthy, Ruth MacRedmond

**Affiliations:** 1 Faculty of Medicine, University of British Columbia, Vancouver, BC Canada; 2 Centre for Heart Lung Innovation, University of British Columbia, Vancouver, BC Canada; 3 Department of Respiratory Medicine, St. Vincent’s University Hospital, Dublin 4, Ireland; 4 School of Medicine, University College Dublin, Dublin 4, Ireland; 5 St. Paul’s Hospital, Vancouver, BC Canada; 6 Division of Critical Care, University of British Columbia, Vancouver, Canada; 7 Unit 8B, Providence Building, St. Paul’s Hospital, Vancouver, BC V6Z 1Y6 Canada

To the editor,

We read the recently reported study by Drs Martín-Fernández et al. [1] with interest given the growing discussion over target oxygenations levels in critical care settings [2]. The authors report the result of a secondary analysis of a prospective cohort of 454 adult patients admitted to surgical ICU with a diagnosis of post-surgical sepsis or septic shock, requiring invasive mechanical ventilation (IMV). The authors sought to compare outcomes between groups stratified on the basis of the partial pressure of arterial oxygen (PaO_2_) at the time of baseline assessment and concluded that hyperoxemia (defined as a PaO_2_ > 100 mmHg on the day of sepsis onset *and* maintained throughout 48 h) was associated with shorter duration of IMV, prolonged survival time, and reduced odds of death at 90 days, when compared to a baseline paO_2_ of < 100 mmHg (“normoxemia”). We feel, however, that certain aspects of the study design and data analysis warrant greater clarification to facilitate drawing accurate conclusions.

Given the observational nature of this analysis, great care must be given to accounting for confounders. When comparing the baseline demographics (Additional file 1: Table S1), significant differences were demonstrated, many of which do not appear to have been included in the final multivariate models, including the significantly higher rate of chronic lung disease (22.3% vs 12.5%, *p* = 0.005), respiratory sepsis (73% vs 33%, *p* < 0.001) and septic shock (84.0% vs 67.1%, *p* < 0.001) in the normoxemia group. Moreover, details regarding the differences in the proportion and severity of ARDS between groups are lacking. Based on the median PaO_2_/FiO_2_ ratios (157.64 vs 278 mmHg, *p* < 0.001), the data would suggest that most patients in the hyperoxia group had at most mild ARDS while those in the normoxia group had at least moderate ARDS (provided other diagnostic criteria were met). Conversely, chronic renal failure was included in multivariate models, despite no significant difference between the stratified groups (20% vs 21%, *p* = 0.66), while data on acute kidney injury, a more significant predictor of negative outcome in ICU than chronic kidney disease, was not presented.

Collectively, these issues are crucial to informed interpretation of the results, given that an association between lung disease and the primary stratification (PaO_2_) was clearly demonstrated in the study, and chronic lung disease and ARDS are known to be a predictors of worse survival outcomes in ICU and post-surgery [3, 4]. Similar effects, specifically for time on ventilator, would be expected to be associated with respiratory sepsis and ARDS compared to other causes. We would suggest that clarification of the selection criteria for co-variates should be provided. Based on the data presented and the outcomes of interest, adjustment for chronic lung disease, respiratory sepsis, shock and ARDS severity would appear to be both statistically and clinically appropriate.

Furthermore, while patients with a PaO_2_ < 100 mmHg were classified as ‘normoxemic’ in this study, this grouping combines severely hypoxemic individuals (with PaO_2_ < 60 mmHg) with normoxemic/moderately hypoxemic individuals. The former represents a wholly different class of risk. Indeed, in the original Fig. 2 (plot of adjusted odds (aOR) of death versus PaO_2_), most of the incresead mortality risk was associated with a PaO_2_ < 60 mmHg, a clearly sub-optimal oxygenation status, and not one that has been entertained in the wider discussion over liberal vs conservative oxygen strategies. Though the authors performed sub-analyses excluding individuals with paO_2_ < 60 mmHg, reporting results consistent with the main analyses, in Fig. 2 no definitive association between improved survival and higher oxygenation was seen above a PaO_2_ of 60 mmHg, especially given the wide confidence intervals displayed as the PaO_2_ passes to > 100 mmHg. Indeed, no difference in the aOR of death was seen at all between a PaO_2_ of ≳ 60 and ≲ 175 mmHg. Again, whether clinically appropriate co-variates were included in the multivariate sub-analyses should be clarified.

Summarily, the results appear to suggest that profound hypoxemia at the onset of sepsis post-surgery is a predictor of poorer outcomes, but how much of this this effect is accounted for by the significant baseline differences is unclear. By the same logic, the conclusion that baseline PaO_2_ > 100 mmHg was “independently” associated with better outcomes is questionable.

Finally, it is important to point out that stratification based on PaO_2_ formed the basis of this post-hoc analysis of an observational study, but to our understanding the same PaO_2_ measurements were not the result of pre-specified oxygen titration strategies, randomized to achieve dichotomized target PaO_2_ ranges. Consequently, though the authors state in the introduction that their study might help to “test the hypothesis that hyperoxemia would improve outcome compared to conservative oxygen therapy in patients with sepsis/septic shock” such a hypothesis cannot be tested by this study design. As such, the author’s assertion that their findings “could have an important clinical relevance for reducing overall mortality in postoperative patients developing sepsis/septic shock” should be interpreted with care.

## Supplementary Information

**Additional file 1**. Baseline characteristics of patients.

## Data Availability

Not applicable.
