# Supplementary material for: Reply to: “Hyperoxemia in postsurgical sepsis/septic shock patients is associated with reduced mortality”
Source: Crit Care. 2022 Jun 9;26:172. doi: 10.1186/s13054-022-03989-z (PMC9185931; doi:10.1186/s13054-022-03989-z)
Supplement: Supplementary file 1 — Additional file 1. Baseline characteristics of patients. [file 13054_2022_3989_MOESM1_ESM.docx]

From: Hyperoxemia in postsurgical sepsis/septic shock patients is associated with reduced mortality [1]

|  | **PaO_2_ ≤ 100 mmHg (1) (*n* = 238)** | **PaO_2_ > 100 mmHg (2) (*n* = 216)** | ***p* value (1 vs. 2)** |
| --- | --- | --- | --- |
| Characteristics | | | |
| Age [years, median [IQR]] | 73 [14] | 72 [16] | 0.69 |
| Male [%, (n)] | 65.5 (156) | 54.6 (118) | 0.028 |
| Comorbidities [% (n)] | | | |
| Chronic cardiovascular disease | 34.9 (83) | 27.8 (60) | 0.09 |
| Chronic respiratory disease | 22.3 (53) | 12.5 (27) | 0.005 |
| High blood pressure | 59.7 (142) | 54.6 (118) | 0.22 |
| Chronic renal failure | 8.4 (20) | 9.7 (21) | 0.66 |
| Chronic hepatic failure | 3.3 (8) | 4.2 (9) | 0.67 |
| Diabetes mellitus | 22.7 (54) | 22.2 (48) | 0.86 |
| Cancer | 27.3 (65) | 39.4 (85) | 0.008 |
| Immunosuppression | 4.6 (11) | 3.7 (8) | 0.61 |
| Obesity | 18.5 (44) | 11.1(24) | 0.024 |
| Surgery type, [% (n)] | | | |
| Abdominal | 60.9 (145) | 76.4 (165) | 0.009 |
| Cardio-thoracic | 2.9 (7) | 3.2 (7) | 0.98 |
| Vascular | 6.3 (15) | 3.2 (7) | 0.07 |
| Urological/renal | 2.1 (5) | 2.3 (5) | 0.99 |
| Other | 4.6 (11) | 2.3 (5) | 0.12 |
| Source of infection, [% (n)] | | | |
| Respiratory tract | 32.9 (73) | 16.3 (33) | < 0.001 |
| Abdomen | 50.0 (111) | 61.9 (125) | 0.014 |
| Urinary tract | 3.4 (8) | 4.6 (10) | 0.50 |
| Surgical site | 0.8 (2) | 1.4 (3) | 0.58 |
| Bacteremia | 3.8 (9) | 3.2 (7) | 0.74 |
| Other | 11.3 (27) | 11.6 (25) | 0.96 |
| Microbiology, [% (n)] | | | |
| Gram + | 29.4 (70) | 29.2 (63) | 0.95 |
| Gram − | 38.2 (91) | 31.0 (67) | 0.11 |
| Fungi | 16.4 (39) | 10.6 (23) | 0.08 |
| Measurements at diagnosis [median [IQR]] | | | |
| PaO_2_ (mmHg) | 74.15 [25] | 134 [45] | < 0.001 |
| FiO_2_ | 0.50 [0.05] | 0.50 [0] | 0.47 |
| PaO_2_/FiO_2_ ratio (mmHg) | 157.64 [73.45] | 278 [108.50] | < 0.001 |
| PaO_2_ at 48 h (mmHg) | 71.50 [25] | 131 [45] | < 0.001 |
| Total bilirubin (mg/dl) | 0.89 [1.06] | 0.75 [0.82] | 0.12 |
| Glucose (mg/dl) | 168 [76.15] | 171 [75.25] | 0.82 |
| Creatinine (mg/dl) | 1.90 [2.51] | 1.51 [1.73] | 0.001 |
| Na (mmol/L) | 137 [6] | 136 [5] | 0.024 |
| K (mmol/L) | 4 [1.1] | 4 [1] | 0.54 |
| Platelet count (cells/mm^3^) | 173,000 [155500] | 197,000 [184000] | 0.014 |
| Lactate (mmol/L) | 2.66 [2.17] | 2.20 [2.20] | 0.08 |
| Procalcitonin (ng/ml) | 5.50 [16.28] | 5.05 [23.41] | 0.61 |
| C-Reactive Protein (mg/L) | 250.55 [176.50] | 232 [155.40] | 0.039 |
| White Blood cells (cells/mm^3^) | 13,660 [12295] | 12,955 [10521] | 0.06 |
| Lymphocytes (cells/mm^3^) | 961.65 [988.14] | 767.70 [901.52] | 0.003 |
| Neutrophils (cells/mm^3^) | 11,727.31 [11263.94] | 11,308.80 [9629.29] | 0.16 |
| Urgent surgery | 66.4 (158) | 80.1 (173) | 0.001 |
| SOFA score | 9 [3] | 7 [5] | < 0.001 |
| APACHE II score | 16 [7] | 14 [7] | < 0.001 |
| Time course and outcome | | | |
| Length of hospital stay [days, median (IQR)] | 25 [24] | 23.50 [27] | 0.97 |
| Length of ICU stay [days, median (IQR)] | 8 [13] | 5 [9] | < 0.001 |
| Length of mechanical ventilation [days, median (IQR)] | 2 [8] | 1 [4] | < 0.001 |
| Sepsis [% (n)] | 16.0 (38) | 32.9 (71) | < 0.001 |
| Septic shock [% (n)] | 84.0 (200) | 67.1 (145) | < 0.001 |
| Mortality at 90 days [% (n)] | 37.0 (88) | 25.5 (55) | 0.008 |

Continuous variables are represented as median and interquartile range (IQR); categorical variables are represented as percentages (%) and number (n). ICU: intensive care unit; SOFA: Sequential Organ Failure Assessment Score

1. Martín-Fernández M, Heredia-Rodríguez M, González-Jiménez I, Lorenzo-López M, Gómez-Pesquera E, Poves-Álvarez R, Álvarez FJ, Jorge-Monjas P, Beltrán-DeHeredia J, Gutiérrez-Abejón E, Herrera-Gómez F, Guzzo G, Gómez-Sánchez E, Tamayo-Velasco Á, Aller R, Pelosi P, Villar J, Tamayo E. Hyperoxemia in postsurgical sepsis/septic shock patients is associated with reduced mortality. *Critical care (London, England)* 2022: 26(1): 4-4.
