# Supplementary material for: Reply to: “Hyperoxemia in postsurgical sepsis/septic shock patients is associated with reduced mortality”
Source: Crit Care. 2022 Jun 9;26:172. doi: 10.1186/s13054-022-03989-z (PMC9185931; doi:10.1186/s13054-022-03989-z)
Supplement: Related texts

# Reply: Authors’ response

**Authors:** Marta Martín-Fernández, Eduardo Tamayo, Jesús Villar

Dear Editor,

We would like to thank to Dr. Franciosi and colleagues for their interest and comments about our article [1]. As they mentioned, in recent years, there has been a great interest on knowing the optimal levels of oxygenation in critically ill patients. Summary data from meta-analyses seem to indicate that hyperoxemia worsens outcome [5]. However, according to a recent systematic review with data from eight trials (4415 participants), the authors did not find differences in mortality (OR 0.95, 95% CI 0.74–1.22) between high or low oxygen targets in mechanically ventilated adults. However, the high heterogeneity and the overlapping target ranges limit its validity and clinical relevance, and calls for urgent further research to define optimal oxygen therapy targets [6]. There are controversial findings in patients with septic shock. For example, in the ICU-ROX trial, no differences were found when comparing conservative versus liberal oxygen therapy [7]. In addition, the use of hyperoxic therapy reduced the risk of surgical site infections in colorectal surgery [8], probably due to the bactericidal activity of neutrophils mediated by oxidative killing, a potent mechanism that depends on the production of superoxide radicals from molecular oxygen.

We sought to review our historical series of septic patients focusing on the PaO_2_ values at the time of sepsis diagnosis and within the first 48 h after diagnosis. We observed that patients with PaO_2_ > 100 mmHg had lower 90-day mortality (25.5% vs. 37.0%, *p* = 0.008). Also, the potential association between PaO_2_ levels and risk of 90-day mortality in our study was further evaluated by using a multivariate logistic regression analysis. Using the variables in Table 1, a univariate regression analysis was performed for 90-day mortality. Variables showing a *p* value < 0.1 with no collinearity were included as adjustment variables in the multivariate analysis. Chronic cardiovascular disease, chronic respiratory disease, cancer, obesity, urgent surgery and platelet count showed a *p* value > 0.1 while bilirubin, creatinine, lactate, neutrophils and white blood cells showed collinearity with APACHE II score. C-reactive protein showed collinearity with procalcitonin. The variables considering “time function”, such as mechanical ventilation, ICU and hospital stay, were considered not to be mixed with variables of admission day. Thus, Table 1 just compares patients with PaO_2_ ≤ 100 mmHg versus PaO_2_ > 100 mmHg.

Furthermore, our study only showed an association between mortality and PaO_2_ ≤ 100 mmHg. We thought that this finding is clinically relevant and should be validated in a clinical trial fashion design, as stated in our paper.
